# Clinico-Morphological Features and Immunohistochemical Profile of a Rare Case of Three Synchronous Primary Malignancies in the Female Genital Tract

**DOI:** 10.3390/reports7010014

**Published:** 2024-02-17

**Authors:** Mădălina Boșoteanu, Raluca Ioana Vodă, Gabriela Izabela Balţǎtescu, Mariana Aşchie, Luana-Andreea Nurla, Cristian Ionuţ Orǎşanu

**Affiliations:** 1 Clinical Service of Pathology, “Sf. Apostol Andrei” Emergency County Hospital, 900591 Constanţa, Romania; mbosoteanu@yahoo.com (M.B.); gabrielabaltatescu@yahoo.com (G.I.B.); aschiemariana@yahoo.com (M.A.); critian.ionut@gmail.com (C.I.O.); 2 Department of Pathology, Faculty of Medicine, “Ovidius” University of Constanţa, 900527 Constanța, Romania; 3 Center for Research and Development of the Morphological and Genetic Studies of Malignant Pathology-CEDMOG, “Ovidius” University of Constanţa, 900591 Constanța, Romania; 4 “Elias” Emergency University Hospital, Dermatovenerology Department, 011461 Bucharest, Romania; luana.bosoteanu@gmail.com; 5 Institute of Doctoral Studies, Doctoral School of Medicine, “Ovidius” University of Constanţa, 900573 Constanţa, Romania

**Keywords:** cervix, ovary, p53, prognosis, uterus

## Abstract

(1) Background: Synchronous tumors are defined as tumors that occur at the same time, appearing within 2 months, according to the Surveillance Epidemiology and End Results Program, with a frequency of tertiary tumors of 0.5%. The purpose of this presentation is to report a case of three synchronous tumors of the female genital tract, given the fact that it represents a challenge both therapeutically and in demonstrating that the malignant lesions are completely different from each other. (2) Methods: We report the case of a 45 year-old patient diagnosed with three synchronous tumors developed in the genital tract: clear-cell ovarian carcinoma, uterine endometrioid adenocarcinoma, and cervical adenosquamous carcinoma. (3) Results: Total hysterectomy with bilateral anexectomy was performed and accompanied by a biopsy of the greater omentum. The evolution of the patient was favorable during chemotherapy, but she died two weeks after the completion of this treatment, from a cause secondary to the adverse effects determined by it, namely, severe thrombopenia which caused a massive lower digestive hemorrhage. (4) Conclusions: This case demonstrates the maximum importance of the involvement of adjuvant diagnostic techniques, especially when it comes to a diagnostic challenge with direct implications in the subsequent therapy of the patient.

## 1. Introduction

Synchronous tumors are defined, according to the North American Association of Central Cancer Registries, as tumors that occur at the same time, while the Surveillance Epidemiology and End Results Program annexes their emergence in a 2-month interval to the definition [1]. The frequency of double primary tumors is 3–5%, whereas tertiary tumors account for 0.5%, and a fourth primary tumor has a frequency of 0.3% [1]. Usually, these tumors appear during the premenopausal period in nulliparous patients and those with comorbidities such as obesity [2]. The most frequent associations of synchronous tumors are those located at the level of the uterine body (without the cervix) and the ovary, while the association of synchronous tumors at the level of the cervix and at the level of the upper half of the uterus is rare [3]. Cases of synchronous tumors occurring in the uterine body, cervix, and ovaries have been much less documented [3]. The role of exposure to certain carcinogens has been described in the occurrence of synchronous tumors in organs with the same embryological origin, such as the uterus and cervix. However, the etiology of synchronous tumors, regardless of common embryological origin, is not known [4]. These patients have a better prognosis compared to those with metastatic disease located in the same organs, especially since there is no clear evidence that any post-therapeutic follow-up strategy would improve survival rates [4].

The purpose of this presentation is to report in the literature a rare and interesting case regarding three synchronous tumors of the female genital tract. Such cases represent a challenge both in the therapeutic decision and in performing the differential diagnosis, in order to demonstrate that the malignant lesions are completely different from each other and that they do not represent the metastasis of any of the tumors to the rest of the reproductive organs.

## 2. Detailed Case Description

### 2.1. Clinical Presentation

**Figure 1:**
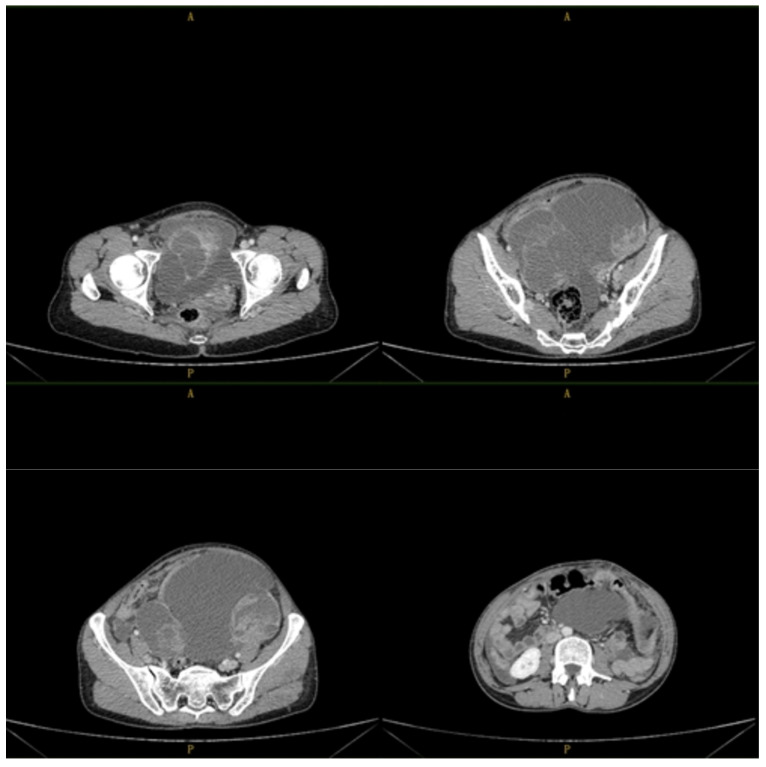
Abdominal-pelvic aspects of ovarian tumor.

A 45-year-old female patient, with no significant personal pathological gynecological history, was hospitalized for abnormal vaginal bleeding and abdominal pain. The clinical examination revealed an increase in the consistency of the cervix, which was painless on palpation; a painful tumor of medium consistency, mobile on the overlying and underlying structures, was palpated in the left iliac fossa. The unenhanced, as well as contrast CT examination of the abdomen and pelvis, detected a tumor of 19/18/17 cm, with projection on the uterine and ovarian areas; an irregular outline and native heterodense structure, associated with non-iodophilic liquid areas and iodophilic tissue areas, were described (Figure 1).

Also, a hypoattenuating, hypoechoic mass in the endometrial cavity was identified. Additionally, a small amount of localized perihepatic fluid collection and diffuse densification of the peritoneum were identified. Based on these descriptions, the presence of an ovarian cystic tumor and endometrial proliferation was concluded. Blood tests displayed mild anemia and elevated levels of CA-125 (1200 U/mL).

Secondary to abnormal vaginal bleeding, it was decided to perform an endometrial biopsy, which highlighted a malignant epithelial proliferation with solid and glandular architecture, and marked nuclear pleomorphism, indicating the diagnosis of endometrioid adenocarcinoma. Consequently, the patient underwent a surgical intervention: total hysterectomy with bilateral adnexectomy, pelvic and para-aortic node dissection, and infracolic omentectomy. Intra-operatively, a left ovarian nodular lesion was observed, imprinting the uterus and the contralateral ovary. There were no macroscopical lesions on the surface of the omentum. A sample of the intra-abdominal effusion was collected for cytological examination. Also, a frozen section was performed from the ovarian tumors, revealing a malignant epithelial tumor. The patient was discharged one week after the surgery, in good general health, with the aim of establishing a properly tailored oncological treatment. 

One month postoperatively, the patient started chemotherapy with six cycles of carboplatin (AUC 7.5/3 weeks, intravenously) and paclitaxel (175 mg/m^2^, intravenously, every 3 weeks). The evolution was favorable during the adjuvant treatment, but the patient died two weeks after the completion of the oncological treatment due to a massive lower intestinal hemorrhage, which developed on the background of a severe thrombocytopenia (75.3 × 103/μL; normal values between 150 × 103/μL and 450 × 103/μL).

### 2.2. Pathological Findings

**Figure 2:**
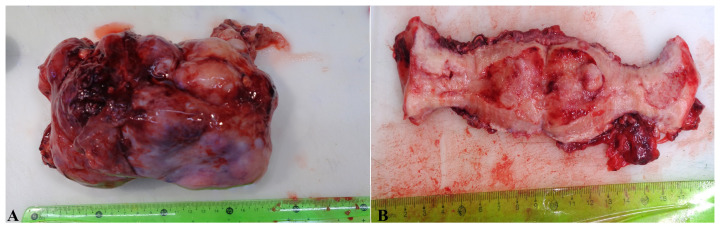
Macroscopical characteristics of (**A**) tumor-transformed ovary, with a maximum diameter of 19 cm; (**B**) cervix with parietal thickening and a vegetative lesion in the uterine cavity.

**Figure 3:**
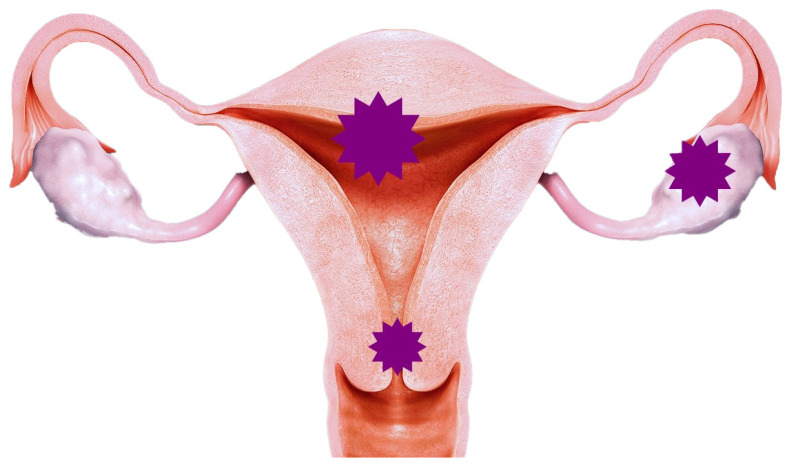
Schematic representation of the location of the three tumors—their location is marked with asterisks.

The macroscopic examination showed a reddish tumoral mass in the left ovary, with friable parietal exophytic lesions and inner solid and cystic areas. The contralateral ovary presented an irregular surface. The cervix revealed a thickening of the endocervical wall, with the presence of necrosis areas. The uterine cavity was occupied by a fungating lesion without an apparent involvement of the uterine wall and the endocervix (Figure 2). The location of the three cancers is better highlighted in Figure 3.

**Figure 4:**
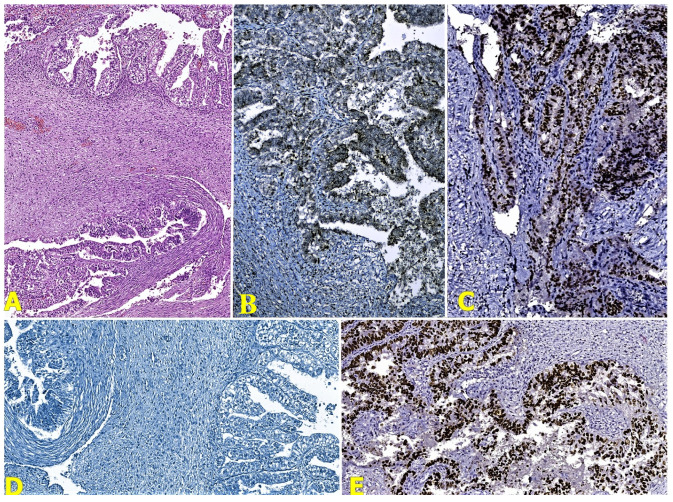
(**A**) Microscopical features of the clear-cell ovarian carcinoma (HE stain, Ob ×50); (**B**) Napsin-A stain (Ob ×100); (**C**) ER stain (Ob ×100); (**D**) WT1 stain (Ob ×100); and (**E**) p53 stain (Ob ×100).

The cytological examination of the abdominal effusion revealed a hemorrhagic and inflammatory fluid with rare clustered epithelial cells with cyto-nuclear atypia. The microscopic examination of the ovarian tumor showed a malignant epithelial proliferation with papillary, glandular, and alveolar patterns, marked cyto-nuclear pleomorphism, and areas composed of malignant cells with clear cytoplasm (Figure 4a). The invasion of the angio-lymphatic spaces and capsule rupture before surgery were associated. The right ovary highlighted the same type of tumor cells. Also, it determined the parametrial invasion. 

**Figure 5:**
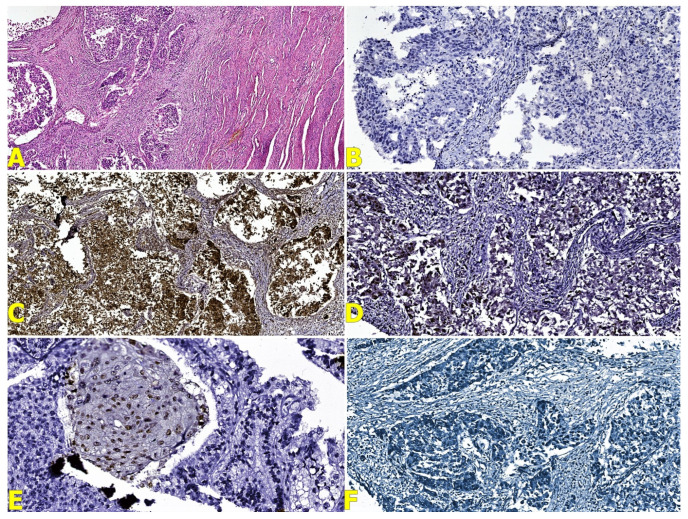
(**A**) Microscopical features of the adenosquamous carcinoma of the cervix (HE stain, Ob ×50); (**B**) ER stain (Ob ×100); (**C**) p16 stain (Ob ×100); (**D**) p53 stain (Ob ×100); (**E**) p63 stain (Ob ×200); and (**F**) WT1 stain (Ob ×100).

The microscopic evaluation of the cervix showed lesions compatible with adenosquamous carcinoma, made up by atypical glandular and squamous cell populations with central keratinization, infiltrating the cervical stroma and affecting the angio-lymphatic spaces; neurotropism was also present (Figure 5a). 

**Figure 6:**
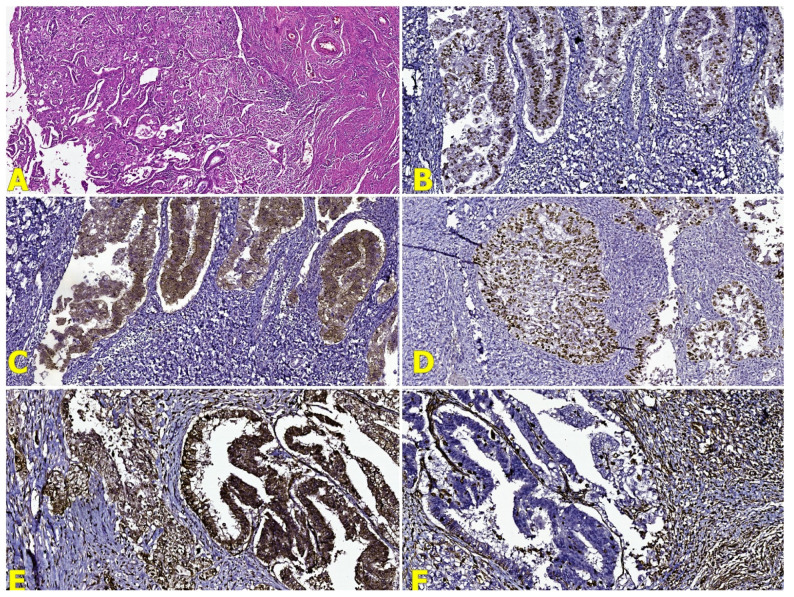
(**A**) Microscopical features of the high-grade endometrioid carcinoma (HE stain, Ob ×50); (**B**) ER stain (Ob ×100); (**C**) p16 stain (Ob ×100); (**D**) p53 stain (Ob ×100); (**E**) Vimentin stain (Ob ×100); and (**F**) WT1 stain (Ob ×100).

The depth of invasion of this tumor was limited to the level of the cervical stroma, having a value of 10 mm. The fungating mass diagnosed in the uterine cavity had a microscopic appearance suggestive of a poorly differentiated endometrioid adenocarcinoma (G3), with nuclear grade 3 (FIGO) and architectural grade 2 (FIGO); furthermore, it showed an infiltrative behavior in the inner half of the myometrium and positive angiotropism (Figure 6a). No lymph node metastasis and no omentum involvement were identified. Microscopically, no continuity or transition pattern was identified between the endometrial tumor and the cervical tumor.

The final histopathological diagnoses comprised clear-cell ovarian carcinoma (pT2a, FIGO IIA), cervical adenosquamous carcinoma (pT1b2, FIGO IB2), and uterine endometrioid carcinoma (pTIa, FIGO IA). This extremely rare type of neoplastic association raised a diagnostic challenge that imposed an immunohistochemical evaluation, aiming to exclude the presence of a metastasis originating from one of the tumoral entities. 

Considering the complex differential diagnosis process, an immunohistochemical evaluation of the three lesions was performed using seven biomarkers; following this thorough examination, a series of essential findings emerged (Table 1).

Corroborating the previously mentioned results, the diagnosis of three primary synchronous malignancies in the same patient was established, including a clear-cell ovarian carcinoma (Figure 4b–e), adenosquamous cervical carcinoma (Figure 5b–f), and high-grade endometrioid carcinoma (Figure 6b–f), with the exclusion of potential metastatic lesions.

## 3. Discussion

Multiple primary cancers represent 1–2% of all gynecological malignancies in female patients [5]. Certain studies suggest that synchronous primary cancers exhibit a better prognosis compared to the cases of a single primary malignancy that has metastasized [6]. 

The etiology of this tumoral category presumably involves genetic predisposition, immunological status, and exposure to carcinogenic factors [5]. In our case, the overexpression of p53 was observed in two out of three tumors, demonstrating its genetic mutation status.

P53 is a nuclear transcriptional regulator engaged in a variety of cellular processes, such as the activation of the DNA repair proteins, the initiation of apoptosis in case of irreparable DNA defects, and the stoppage of cell division by blocking the cellular division cycle in the G1/S transition phase; additionally, this protein has a documented role in senescence, genomic stability, and metabolic homeostasis [7,8]. It is activated by various signals (e.g., DNA defects, oncogene expression, or ribonucleotide depletion); therefore, this genetic dysfunction is observed in over 50% of malignancies [7,8]. Genetic aberrations involved in carcinogenesis encompass specific changes in gene sequence (e.g., mutations), gene amplifications or deletions, genetic rearrangements, and chromosome translocations [7].

Ovarian epithelial carcinomas are the gynecological malignancy with the highest mortality and the fifth leading cause of cancer death in women. In recent decades, there has been a 14% decrease in the incidence and death rate from cancer for this tumor category [7]. These malignant neoplasms are classified into subtypes I and II based on clinical, histopathological, and molecular aspects [8]. Subtype I is represented by low-grade serous carcinoma, endometrioid carcinoma, and clear-cell carcinoma, and in subtype II, there are high-grade serous carcinoma, carcinosarcoma, and undifferentiated carcinoma [9]. Clear-cell carcinoma represents 5–6% of ovarian carcinomas, having pathological characteristics that do not allow exact framework in one of the two subtypes, being considered a high-degree tumor from the time of diagnosis [10]. However, the prognosis of clear-cell ovarian carcinoma is unfavorable compared to other carcinomas, such as serous or mucous, with a 5-year survival rate of 27% [11].

Genetic alterations of p53 are rarely reported in clear-cell ovarian carcinomas [12]. There are many studies that have reported different frequencies of p53 mutations in these types of carcinomas. In the study carried out by Wenbin Xiao on 26 clear-cell ovarian carcinomas, the presence of p53 overexpression was observed in 7.7% of cases [13]. Also, DeLair D studied 155 clear-cell carcinomas, which he divided in two groups depending on the presence of typical pathological characteristics and the most rare ones, in the last group observed at 24% the presence of p53 mutant type [14]. According to Nakano T et al., the intranuclear accumulation of p53 was observed in 15 of 31 cases. The positive immunoreaction of p53 and increased proliferative activity were associated with an unfavorable/poor prognosis; the response rate to chemotherapeutic treatments such as those based on platinum was much lower in patients with positive p53 (20%) than in those whose reaction was negative (66.7%), implying that the former are chemoresistant [15]. Radio-resistance in relation to p53 mutations was studied by Langland GT et al. on 16 ovarian cell lines, noting that the presence of the p53 mutation is present in most radio-resistant cell lines [16].

WT1 (Wilms Tumor 1) expression is found in various cell types within the gynecological tract, such as the surface epithelial cells of the ovaries and fallopian tubes, granulosa cells, myometrium, and endometrial stromal cells. In addition, the immunohistochemical expression of WT1 is highly valuable in the field of gynecological pathology. It plays a crucial role in diagnosing ovarian serous carcinoma, encompassing both high-grade and low-grade histotypes. Furthermore, it aids in efficiently distinguishing carcinomas originating from the ovaries from carcinomas originating elsewhere in the body [17].

After binding to their subtypes (ER α and ER β), estrogens stimulate various physiological actions, including cell survival and proliferation [18]. In terms of immunohistochemistry, ovarian clear-cell carcinoma typically shows strong positivity for hepatocyte nuclear factor 1β, while it is consistently negative for estrogen receptor (ER), progesterone receptor (PR), and WT-1 in over 95% of cases [19,20].

Napsin-A possesses a strong potential as a reliable immunohistochemical marker for clear-cell carcinomas of the gynecologic tract. It exhibits expression rates ranging from 56% to 93% in endometrial clear-cell carcinomas, 82% to 93% in ovarian clear-cell carcinomas, and 70% to 71% in endocervical clear-cell carcinomas [21]. 

AMACR, also known as alpha-methylacyl-CoA racemase, plays a vital role in lipid metabolism as it functions within both the mitochondria and peroxisomes. The expression of this maker is widely used for the diagnosis of prostate adenocarcinoma and papillary renal cell carcinoma [22]. In ovarian clear-cell carcinoma, AMACR is significantly overexpressed, surpassing other forms of epithelial tumors [23].

In the present case, the immunohistochemical expression of the studied markers (p53, WT1, ER, PR, NapsinA, and AMACR) confirms the diagnosis of clear-cell ovarian carcinoma, excluding the possibility of metastasis from the other identified malignant tumors.

Endometrial carcinoma is classified into two types: type I is represented by low-grade endometrioid carcinomas, estrogen-dependent, with frequently indolent clinical evolution, and type II includes clinically aggressive, non-endometrioid carcinomas, which are not related to estrogen stimulation and include serous and clear-cell carcinomas [24]. P53 dysfunction in endometrial cancers consists in its mutation. With a frequency of only 10–40% in endometrioid carcinomas, missense-like mutations cause nuclear protein accumulation, resulting in immunohistochemical overexpression [8]. 

Mi Jin Kim and Dong Suk Kim studied the immunohistochemical expression of p53 in 25 cases of endometrioid carcinomas, only 6 (24%) of which were positive and significantly correlated to the histological grade, nuclear grade, and myometrial invasion [25]. In the study conducted by Opric D et al., examining 97 patients with endometrial carcinoma, 72 cases (74.2%) were diagnosed as the endometrioid type; the p53 expression was positive in 13.8% of the total number of cases, revealing that the frequency of p53 overexpression proportionally increases in conjunction with the histological grade [26]. In addition, according to Hussein YR and Soslow RA, the p53 mutation was reported in 20–30% of the cases of FIGO3 endometrioid carcinoma, a notion also applicable to the present case [27]. 

Endometrioid carcinomas frequently display sporadic p16-positive staining, which typically manifests as a mosaic pattern but can also be quite extensive [28]. 

According to the literature, in the genital area, vimentin is positive in most endometrial carcinomas, carcinosarcomas, and uterine mucinous carcinomas [29]. The results of our case are consistent with these data, showing an immunoreaction of this marker in endometrioid endometrial carcinoma.

In patients with endometrial carcinoma, the presence of the simultaneous negative immunoreaction for ER, PR, and HER2 is found in 15–20% of cases. This characteristic is associated with unfavorable pathological characteristics, including high-risk histological type, a deeper invasion of the myometrium, and higher histological grade and clinical staging. Moreover, their presence is also linked to shorter survival rates [30]. The loss of both estrogen receptor and progesterone receptor expression is a powerful indicator of a negative prognosis. This applies not only to low-grade endometrial carcinomas but also demonstrates the potential to predict lymph node metastases and the risk of recurrence [31]. The above idea is also supported by the study of Waqar S. et al., in which the majority of high-grade endometrial cases are ER- and PR-negative [32].

These characteristics of hormonal receptors are also found in this case, being one of the peculiarities, in accordance with the data presented in the literature.

Invasive adenosquamous carcinoma is a relatively scarce histological subtype, classified by the WHO as a different entity from the pure squamous and glandular malignancies; this distinction was based on morphology, on the association with HPV, and on its specific clinical evolution. The study published by Stolnicu S. et al. evaluated the immunohistochemical expression of 59 cases of cervical adenosquamous carcinoma. P53 status was examined in 25 cases, among which 2 (8%) displayed positivity, while p16 expression was present in 18 out of 25 cases (72%), as opposed to 82.6% of cases where HPV was detected by in situ hybridization [33]. These data show the association of HPV infection with the occurrence of adenosquamous carcinoma, thus also illustrating the involvement of p53 mutation.

Concerning the survival rates, Freier CP et al. studied 248 cases of cervical carcinoma and subsequently observed the association of the p53 mutation with a superior 5-year and 10-year survival prognostic, compared to those with negative p53 immunoexpression [34]. The aforementioned study also showed the absence of post-radiotherapy changes in the p53 levels, suggesting that the degree of p53 expression could be a useful biomarker for predicting the therapeutic response [34]. Contrastingly, in the study carried out by Gbadegesin MA et al., cervical carcinoma cases with an accumulation of p53 mutations presented an increased severity [35].

Reports indicate that p16 demonstrates oncogenic properties in cell lines of cervical carcinoma. This suggests that p16 serves not only as a diagnostic marker for cervical neoplasia but also plays a crucial role in the survival of cervical carcinoma cells [36].

The TP63 gene, located at chromosome 3q28, encodes tumor protein 63 (p63), a transcription factor belonging to the p53 gene family. The activity of numerous genes responsible for the growth and development of the ectoderm and its derived structures and tissues is regulated by p63 [37].

The study conducted by Steurer S et al. reveals that squamous cell carcinomas, regardless of their origin, are the most frequently observed p63-positive cancers. In some of these tumors, p63 neo-expression was clearly associated with focal squamous cell differentiation. Certain tumor types are commonly associated with the presence of squamous elements. Notably, these tumors show a range of p63 positivity between 10% and 25%. This group includes endometroid cancer, malignant mixed Mullerian tumors of the uterus, as well as ovarian, pancreatic, and cholangiocellular carcinomas [38].

In the present case, p63 immunoreaction at the level of adenosquamous carcinoma confirmed the presence of the squamous component and thus confirmed the diagnosis.

The uniqueness of this case consists in the association of the three malignant tumor types (clear-cell ovarian carcinoma, endometrioid endometrial carcinoma, and cervical adenosquamous carcinoma). Until now (1982–2023), only 18 cases of triple synchronous tumors of the female reproductive system have been identified in the specialized literature, but none with this combination [2,3,4,5,39].

The key message of this work is the usefulness and special importance of the correct and complete immunohistochemical examination. This has a bivalent role, both for establishing a diagnosis of certainty, and for making a differential diagnosis between pathological tumor entities, especially when it comes to cases that represent a challenge for pathologists. The work completes and updates the existing literature through the uniqueness of the association of the three types of malignant tumors, namely, ovarian clear-cell carcinoma, endometrioid endometrial carcinoma, and cervical adenosquamous carcinoma, a case that contributes to the enrichment of knowledge both in the medical spectrum (pathologists, gynecologists, and oncologists), and in the field of research.

## 4. Conclusions

Given the rare development of triple synchronous tumors and their poorly elucidated etiopathogenesis, the present case supports the theory that the exposure of tissues with the same embryological origin (uterus and cervix) to certain carcinogens underlies the occurrence of these malignancies. The infrequent case of three synchronous tumors (clear-cell ovarian carcinoma, uterine endometrioid adenocarcinoma, and adenosquamous cervical carcinoma) developed in organs evolving from the same germ layer, adjoined to the immunohistochemical overexpression of p53 with positive reaction in over 80% of the tumoral nuclei, demonstrates the uttermost importance of immunohistochemical technique involvement as a means to achieve a rigorous diagnostic approach. 

## Figures and Tables

**Table 1:** Immunohistochemical evaluation findings of the presented case.

Immunohistochemical Markers (Clone)	Results		
	Clear-Cell Ovarian Carcinoma	Uterine Endometrioid Adenocarcinoma	Cervical Adenosquamous Carcinoma
P53 (EP9)	Over-expression pattern	Over-expression pattern	Wild-type pattern
P16 (BC42)	Negative reaction	Intense positive nuclear reaction with mosaic pattern	Intense diffuse positive nuclear reaction
P63 (4A4)	Negative reaction	Negative reaction	Diffuse positive nuclear reaction in the squamous component
WT1 (rWT1/857)	Negative reaction	Negative reaction	Negative reaction
ER (SP1)	Intense positive nuclear reaction in tumoral glands	Intense positive nuclear reaction in tumoral glands	Negative reaction
Napsin A (TMU-Ad 02)	Intense focal positive cytoplasmic reaction	Negative reaction	Negative reaction
Vimentin (SP20)	Negative reaction	Intense diffuse positive cytoplasmic reaction	Negative reaction
AMACR (13H4)	Intense diffuse positive cytoplasmic reaction	Negative reaction	Negative reaction
PR (16)	Negative reaction	Negative reaction	Negative reaction

## Data Availability

The raw data supporting the conclusions of this article will be made available by the authors on request.
